# Dehydroepiandrosterone (DHEA) and Dehydroepiandrosterone Sulfate (DHEA-S) in Broiler Plasma: Analytical Validation of Immunoassays and Comparative Overview Across Avian Species

**DOI:** 10.3390/ani16131990

**Published:** 2026-06-27

**Authors:** Laura Menchetti, Olimpia Barbato, Giovanni Ricci, Marco Gobbi, Roberta Stocchi, Seyedalireza Kasaiyan, Renzo Galli, Luca Todini

**Affiliations:** 1 Scuola di Bioscienze e Medicina Veterinaria, Università di Camerino, Via della Circonvallazione 93/95, 62024 Matelica, Italy; roberta.stocchi@unicam.it (R.S.); seyedalirez.kasaiyan@studenti.unicam.it (S.K.); luca.todini@unicam.it (L.T.); 2 Dipartimento di Medicina Veterinaria, Università di Perugia, Via San Costanzo 4, 06126 Perugia, Italy; olimpia.barbato@unipg.it; 3 Istituto Zooprofilattico Sperimentale dell’Umbria e delle Marche “Togo Rosati”, Via Maestri del Lavoro, 62029 Tolentino, Italy; m.gobbi@izsum.it; 4 Fileni Agricultural Company, Località Cerrete Collicelli 8, 62011 Cingoli, Italy; r.galli@fileni.it

**Keywords:** animal welfare, steroid hormones, poultry, dehydroepiandrosterone (DHEA), immunoassay validation

## Abstract

This study validated immunoassays for measuring circulating DHEA and DHEA-S in broiler plasma and compared the two biomarkers. A commercially available RIA kit for DHEA and a species-independent ELISA kit for DHEA-S were tested against each other and available literature data. Blood samples were collected from 68 female broilers at slaughter. Most sample concentrations were close to the lower calibration range. DHEA concentrations ranged from 52 to 354 pg/mL, whereas DHEA-S values ranged from 76 to 7320 pg/mL. Both assays showed satisfactory validation results, including good precision (intra- and inter-assay CV ≤ 15%), accuracy (recovery ≥ 99%), linearity (R^2^ > 0.9; run test *p* > 0.1), and parallelism between calibrator curves and sample dilutions (ANCOVA *p* > 0.1). However, the DHEA-S assay showed high cross-reactivity with DHEA (160%), making quantitative DHEA-S determination unreliable; therefore, values should be interpreted as an estimate of combined DHEA/DHEA-S concentrations. Consequently, the higher concentrations measured with the DHEA-S ELISA compared with the DHEA RIA (Bland–Altman bias: −525.8 pg/mL) do not indicate a true predominance of DHEA-S. Nevertheless, both assays may represent useful tools for comparative evaluations among samples, although they do not provide reliable information on the physiological interconversion between the two steroids.

## Simple Summary

Dehydroepiandrosterone (DHEA) and its sulfate ester (DHEA-S) are hormones involved in the physiological response to stress and are attracting growing interest as potential indicators of animal welfare. In poultry, however, information on these biomarkers is still very limited, and validated methods for their measurement are lacking. This study evaluated two immunoassays for measuring DHEA and DHEA-S in the plasma of female broiler chickens sampled at slaughter. Both methods showed acceptable analytical performance and may be useful for comparative studies among animals or experimental groups. However, the DHEA-S assay showed strong cross-reactivity with DHEA, meaning that measured values likely reflected a combined DHEA/DHEA-S signal rather than DHEA-S alone. In addition, the sensitivity of both assays was close to the lower range of detection for chicken plasma. Despite these limitations, the study provides the first data on circulating DHEA(S) in broilers and represents a preliminary step toward the development of hormonal tools that could support future research on stress physiology and welfare assessment in poultry.

## 1. Introduction

Dehydroepiandrosterone (DHEA) and its sulfate ester (DHEA-S), collectively indicated as DHEA(S), are steroid hormones and metabolic intermediates in sex hormone biosynthesis. The conversion of DHEA into DHEA-S is primarily catalyzed by the sulfotransferase type 2A1 enzyme, while steroid sulfatase is the primary enzyme involved in steroid desulfation [1]. In primates, most of the circulating hormones in the blood are in the sulfated form, DHEA-S, which has a slower clearance rate and a longer half-life than DHEA [2]. Although DHEA-S was historically regarded as an inactive metabolite or storage form of DHEA, it is now recognized as an important circulating reservoir for the peripheral formation of bioactive steroids and may also exert direct biological effects [2]. DHEA(S) originates from steroidogenic organs (adrenals, gonads, brain, placenta), with relative contributions widely variable across different species. In primates and hamsters, the main source of circulating is the zona reticularis of the adrenal cortex, which accounts for 75–90% in humans [3]. In other mammalian species, such as rats, mice, and dogs, DHEA(S) are mainly synthesized by the gonads [4]. In dogs, testes are an important source of DHEA in males, whereas in bitches, DHEA is mainly secreted by the adrenal glands, with limited contribution from the ovaries to circulating hormone levels [5].

Beyond their fundamental role as sex hormone precursors, DHEA(S) have anti-glucocorticoid properties [6,7], exerting anabolic, immune-enhancing, anti-oxidative, anti-inflammatory, and neuroprotective functions [8]. DHEA(S) production during acute stress has been suggested to play a protective role in the stress response, acting as an antagonist to the effects of cortisol [9,10]. In humans, DHEA(S) levels have been associated with anti-aging effects [11], mental health, cognitive performance, mood [12], anxiety, and overall welfare [13,14]. Therefore, DHEA(S) attracted attention as potential biomarkers of the Hypothalamic–Pituitary–Adrenal (HPA) axis activity [4,15], at least in the species in which these hormones are primarily produced by the adrenal glands.

Simultaneously measuring the levels of glucocorticoids and DHEA(S), which exert many opposing biological effects, may be an important indicator of net HPA activity. In humans, the cortisol/DHEA(S) ratio [7] has been proposed as a biomarker associated with vulnerability to psychopathological conditions [16] and resilience [10], has been linked to the development of age-related neurodegenerative diseases [17], and is considered a potential objective indicator of psychological stress [3]. Together, these findings support the potential use of DHEA(S), particularly in conjunction with glucocorticoids, as an indicator of affective state-related responses [7,10], a key challenge in the assessment of welfare in domestic animals [18].

In line with this, in farm animals, the cortisol/DHEA(S) ratio is proposed as one potential biomarker of allostatic load and resilience [4,19,20,21], although knowledge in this area remains rather limited. This growing interest reflects the need for integrative physiological indicators that better capture adaptive capacity and reflect the balance between stress-related and counter-regulatory neuroendocrine processes, thereby contributing to the regulation of different affective dimensions of animal welfare, particularly in intensive production systems, such as poultry.

Plasma DHEA concentrations have been assayed in a variety of wild avian species. Many findings suggest that DHEA is involved in territorial aggressive behavior during the non-breeding season, when the gonads are regressed and gonadal steroids are low. In fact, circulating DHEA can be converted into active sex steroids within the brain [22,23,24]. Moreover, in the avian brain, DHEA plays a neuroprotective role, counteracting the neurodegenerative effects of chronic corticosterone, suggesting also its potential translational use in humans for the treatment of glucocorticoid-related neurodegeneration and stress-related psychiatric illness [25]. In birds, blood concentrations are reported to be very low in comparison with primates, so that the assays available should be sensitive enough to provide valuable results.

To our knowledge, DHEA and DHEA-S have never been assayed in chicken (*Gallus domesticus*) blood. The aims of the present study were to (i) validate and apply commercially available immunoassays for the measurement of DHEA and DHEA-S concentrations in plasma samples collected from broilers at slaughter, (ii) evaluate the agreement between the two measurements, and (iii) provide a comparative overview of circulating DHEA and DHEA-S levels reported in avian species to support the interpretation of the results. To this end, we employed a commercially available RIA kit for DHEA in human serum and plasma (DSL-8900, Immunotech s.r.o., Radiova 1122/1, 102 00 Prague 10, Czech Republic), previously validated with modifications and widely used in avian plasma. For DHEA-S, we evaluated an ELISA kit designed to be species-independent and validated across multiple matrices (DetectX® K054, Arbor Assays, Ann Arbor, MI, USA), which has recently been validated for broiler feather extracts [26]. In addition, available data on circulating DHEA and DHEA-S levels in avian species were collected from the literature to support method optimization and provide a comparative framework for interpreting the present findings.

## 2. Materials and Methods

### 2.1. Animals and Blood Sampling

Blood samples were collected from 68 female broilers at an industrial slaughterhouse, as previously described [27], in 3 different sampling sessions. Animals were from an organic farm, commercial strains “slow growth” (Ranger Classic in sessions 1 and 3, Hubbard Red in session 2), aged 87 d (session 1) or 84 d (sessions 2 and 3), with an overall mean live weight of 3.25 kg. The chickens were manually removed from the cages, suspended on the shackle line supplied with breast support, and stunned by an electrified water bath (THF—Version 4″ 400 V, Bayle SA, La Fouillouse, France) according to Council Regulation (EC) 1099/2009 on the protection of animals at the time of killing. Blood samples were collected by dripping from hanging animals immediately after jugulation in labeled 8 mL tubes containing K2EDTA (GREI455040-1200, Greiner Bio-One Italia, Cassina de Pechi, Italy) and centrifuged (2500 *g* for 15 min) within 2 h. Recovered plasma was aliquoted in 1.5 mL Eppendorf polypropylene tubes, immediately frozen, and stored at −20 °C until assayed (within a few months).

### 2.2. Literature Search and Data Collection

Available data on circulating DHEA and DHEA-S concentrations in avian species were collected through a literature search performed using databases such as PubMed and ScienceDirect. Search terms included combinations of “DHEA”, “DHEA-S”, “DHEA(S)”, “dehydroepiandrosterone sulfate”, “avian”, “bird”, “poultry”, “chicken”, “broiler”, “blood”, “plasma”, and “serum”.

The retrieved data, summarized in Table 1 [25,28,29,30,31,32,33,34,35,36,37,38,39,40,41,42,43,44,45,46,47,48,49,50], were used in the subsequent steps to support method optimization and provide a comparative framework for interpreting the present findings. Notably, only one paper reports DHEA-S plasma concentration in birds [28].

### 2.3. DHEA Determination in Unextracted Plasma Samples by RIA Kit, Modifications, and Extraction Attempts

The RIA kit (DSL-8900, Immunotech s.r.o., Radiova 1122/1, 102 00 Prague 10, Czech Republic) is a competitive binding assay with 125I-labeled DHEA as a tracer, and it is intended to be used for the quantitative measurement of DHEA in human serum and plasma (without extraction). Since physiological concentrations in the blood of birds are reported to be many times lower than in humans, the standard curve generated from the provided calibrators is not suitable for avian samples. In fact, the studies with avian plasma utilized this kit (Table 1) with some modifications (dilution of calibrators 10×, the primary antibody, and the tracer 4×) aimed to improve sensitivity, as first proposed for human saliva by Granger et al. [51]. Dilution of calibrators with PBS (0.1% gel) gave final concentrations of 20, 50, 100, 250, 500, and 1000 pg/mL. Nowadays, the kit has substantially changed (since the year 2020, as answered by the producer, upon request), switching from the double-antibody format to polyclonal antibody (from rabbit)-coated tubes. Obviously, any modification of the antibody (and tracer) concentration cannot be made. Therefore, we just diluted all the calibrators 1:2 with PBS (+0.1% BSA, pH 7.4) and added a lowest point. The resulting standard curve ranged from 80 to 21,000 pg/mL.

In most of the studies reported in Table 1, DHEA was assayed after previous steroid extraction and separation from plasma. Mainly, a very fine and efficient solid-phase extraction followed by separation has been proposed [33,35,42,52], which is a rather time-consuming and expensive procedure. On the other hand, prior to the assay of DHEA, steroid extraction from avian plasma samples by diethyl ether has also been utilized [30,43,44]. Recently, Arbogast et al. [53] compared three extraction methods for DHEA from rhinoceros serum, finding diethyl ether to yield the best results. We carried out the standard procedure routinely managed in our laboratory. Briefly, 2 mL of diethyl ether were mixed with 200 µL of plasma sample, vortexed (at least 1 min), and put at −20 °C until the aqueous phase froze. The upper liquid ether phase was then decanted and left to evaporate completely in a 37 °C water bath. The ether extract was then re-dissolved with 200 µL PBS + BSA and vortexed (at least 1 min). We also tried to extend the vortex steps up to 3 min. Resuspension of dried samples with PBS (+0.1% BSA) was done both without and with 10% absolute ethanol, as suggested by Newman et al. [32], to aid the resuspension of steroids. Anyway, the extraction of plasma samples yielded unsatisfactory results, as the analyte concentrations in all samples were below or slightly above the limit of detection. Therefore, we proceed to assay DHEA directly in whole unextracted plasma samples, as also reported for sparrow [39] and geese [50] plasma. Assays have been performed following the manufacturer’s instructions. CPMs have been read 1 min by the gamma counter HIDEX AMG (Turku, Finland) [and Packard Cobra II (Downers Grove, IL, USA).

The results were calculated using a spline curve fit, with B/B0 on the vertical axis and the log of analyte concentration of the calibrators on the horizontal axis, as suggested in the kit leaflet. Sensitivity, reported by the producer as the limit of detection, is 0.25 ng/mL. By the modifications of Granger et al. [51], the limit of the assay’s sensitivity had been lowered to 4.0 pg/mL. In the present study, the detection limit resulted in 20 pg/mL, calculated by interpolating the mean minus 2 SD cpm of the calibrator zero along the standard curve (2 replicates, mean of 10 assays). For specificity, the manufacturer reports that the antibody used is highly specific for DHEA, and low cross-reactivities were obtained with several steroids (DHEA–sulfate, isoandrosterone, androstenedione, etc.).

### 2.4. DHEA-S Determination in Unextracted Plasma Samples by ELISA Kit and Cross-Reactivity Considerations

The EIA kit (DetectX® K054, Arbor Assays, Ann Arbor, MI, USA) is species-independent and validated for serum, plasma, urine, saliva, dried fecal extracts, and tissue culture media samples. The competitive-binding method uses a microtiter plate coated with an antibody to capture sheep antibodies, a DHEA-S peroxidase conjugate, and a polyclonal antibody to DHEA-S. Microplates were read at 450 nm (Tecan Infinite 200 Pro, Tecan Austria GmbH, Grödig, Austria). For human serum and plasma samples, the manufacturer suggests a minimum dilution of 1:2; however, due to their high concentration, most samples must be diluted at least 1:100 with assay buffer. Preliminary assays gave low values for the chicken samples; therefore, undiluted plasma samples were utilized.

The results were calculated using a 4-Parameter Logistic Curve, with B/B0 on the vertical axis and the log of analyte concentration of the calibrators on the horizontal axis, as suggested. As reported in the kit leaflet, sensitivity and limit of detection were determined as 90.9 pg/mL and 75.6 pg/mL, respectively. Regarding specificity, it is noteworthy that a 162% cross-reactivity is reported for DHEA. In human blood, levels of DHEA are typically between 1 and 0.1% of the DHEA-S concentration; therefore, DHEA will contribute to an increase in measured DHEA-S concentrations of less than 2%. However, corresponding information is not available for avian species. In any case, due to the substantial cross-reactivity with DHEA, the measured signal likely reflects the combined contribution of both DHEA-S and DHEA also in our samples, rather than DHEA-S alone. Therefore, the measured analyte may be more appropriately referred to as “DHEA(S)”. Cross-reactivity with other steroids has been reported as 44.5% for epiandrosterone, 28.4% for androsterone, and 15.2% for androstenedione, while all other tested compounds (n = 13) showed cross-reactivities below 0.5%, most of them below 0.1%.

### 2.5. Analytical Validation and Statistical Analysis

The analytical validation process included the assessment of precision (repeatability and intermediate precision), accuracy (recovery), linearity, and parallelism.

Precision was evaluated in terms of repeatability (intra-assay CV), calculated from six replicates (n = 6) of the same sample within a single assay run, and intermediate precision (inter-assay CV), determined from duplicate measurements of the same sample across five independent assay runs (n = 5).

Accuracy was assessed by recovery rates (RRs), calculated as (observed concentration/expected concentration) × 100, after spiking a pooled plasma sample with known quantities of hormone (n = 4), obtained by adding increasing volumes of the highest calibrator (42 and 60 ng/mL for DHEA RIA and DHEA-S ELISA, respectively).

Linearity and parallelism were evaluated through dilution tests. A pooled sample spiked with the highest calibrator was serially diluted using PBS (RIA) or assay buffer (ELISA). Dilution curves were compared with calibration curves to assess parallelism using semi-logarithmic regression models (with the *Y*-axis log-transformed). Slopes within the common analytical range were compared using Analysis of Covariance (ANCOVA). The F-statistic and corresponding two-tailed *p*-value were used to test the null hypothesis of equal slopes. The coefficient of determination (R^2^) was also reported. Linearity was further assessed using linear regression, plotting expected concentrations against observed concentrations. The degree of linearity was evaluated based on the coefficient of determination (R^2^) and the slope of the regression line, with a slope close to 1 indicating optimal agreement. The Runs test was used to verify deviations from linearity. Descriptive statistics were used to summarize the data as mean, minimum (min) and maximum (max) values, and standard error of the mean (SEM). Data distribution and outliers were assessed using diagnostic plots and the Shapiro–Wilk test.

As hormone concentration data were not normally distributed, the relationship between DHEA and DHEA-S concentrations was assessed using two-tailed Spearman’s rank correlation coefficient (ρ). Correlation coefficients (ρ) and corresponding *p*-values were reported. The strength of the correlation was interpreted as poor if ρ < 0.3, medium if 0.3 ≤ ρ < 0.5, and large if ρ ≥ 0.5. Agreement between concentrations obtained by the DHEA RIA and the DHEA-S ELISA was further evaluated using Bland–Altman analysis. For each sample, the difference between the concentrations obtained by the two assays was plotted against their mean value. The analysis provides the mean difference between paired measurements (bias), where “paired” indicates the two values obtained from the same sample by the two assays, and the 95% limits of agreement, representing the interval within which 95% of the differences are expected to lie.

All analyses were performed using GraphPad Prism (v8.0 for Windows, GraphPad Software, Boston, MA, USA), with a significance level of 0.05.

## 3. Results

### 3.1. Validation of the RIA for DHEA Determination

Plasma DHEA concentration was 112.8 ± 7.8 pg/mL (n = 68 individual animals), with a range (min–max) of 52–354 pg/mL.

Precision has been measured by the repeatability of a pooled sample (390 pg/mL). Intra-assay CV (n = 6 replicates) was 12%, and inter-assay CV (2 replicates in n = 5 assay sessions) resulted in 17%. A control sample provided in the kit (4911 pg/mL) showed an inter-assay CV of 18% (for within-laboratory precision, the coefficients of variation were declared by the manufacturer to be below or equal to 17.3% for serum samples).

The mean RR of different quantities of hormones added to the pooled plasma sample was 118% (Table 2).

**Figure 1:**
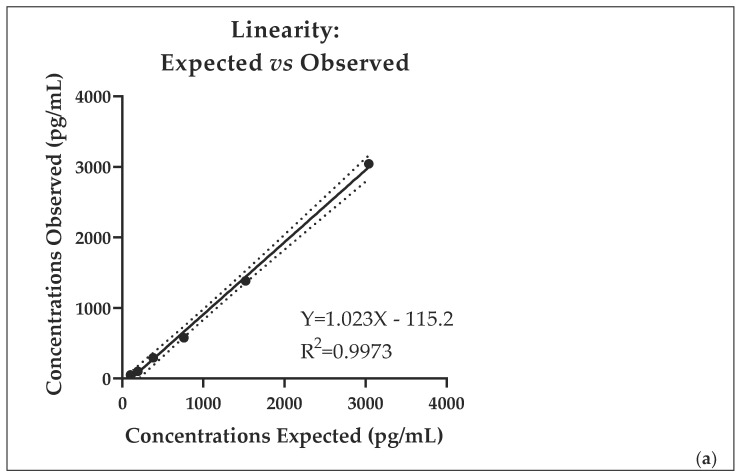

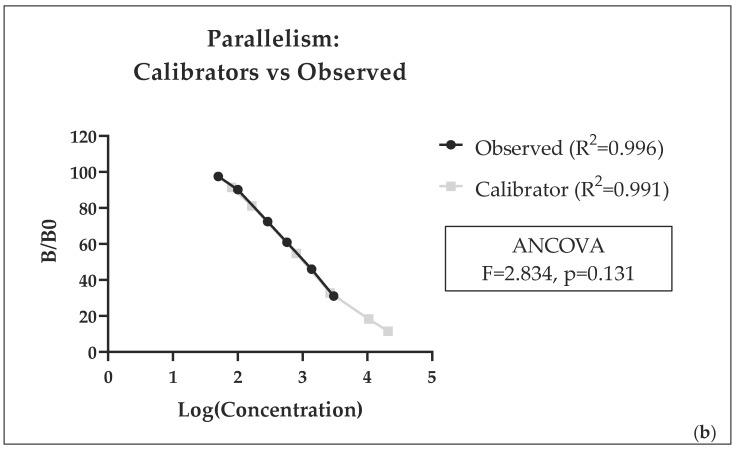
Linearity (panel (**a**); relationship between expected and observed concentrations) and parallelism (panel (**b**); comparison between calibration curve and serial dilutions of a pooled sample) of the RIA for DHEA. In panel (**a**), observed concentrations are shown as black circles. The solid line represents the linear regression, and the dotted lines indicate the 95% confidence bands.

The linearity of the RIA assay was evaluated by comparing expected and observed concentrations across the analytical range. A strong linear relationship was observed, with an R^2^ of 0.9973. The regression equation (Figure 1a) showed a slope close to 1, indicating good agreement between expected and measured concentrations. The Runs test indicated no significant deviation from linearity (*p* = 0.400), confirming the adequacy of the linear model. Overall, these results demonstrate excellent linearity of the assay over the tested range.

Parallelism between the calibration curve and serial dilutions of the pooled plasma sample was assessed using semi-logarithmic regression analysis (Figure 1b). Both the calibrator curve (R^2^ = 0.9911) and the sample dilution curve (R^2^ = 0.996) showed a strong fit. Comparison of the slopes using ANCOVA revealed no significant difference between the two curves (F = 2.834, *p* = 0.131), supporting the assumption of parallelism. This indicates that the assay accurately measures endogenous hormones without significant matrix interference.

### 3.2. Validation of the ELISA for DHEA-S Determination

Ten samples (15%) were below the limit of detection and were treated as missing values because their actual concentrations could not be reliably quantified. To avoid introducing bias through the assignment of arbitrary values, these samples were excluded from quantitative analyses. In the 58 remaining samples, plasma DHEA-S concentrations were 642.8 ± 147.6 (mean ± SEM) with a range (min–max) of 76–7320 pg/mL.

Precision (measured by repeatability) resulted in intra-assay CV 10% (n = 6 replicates, mean 242 pg/mL) and inter-assay CV 15% (2 replicates in n = 5 assay sessions). The mean RR of different quantities of hormone added to the pooled plasma sample was 99% (Table 3).

**Figure 2:**
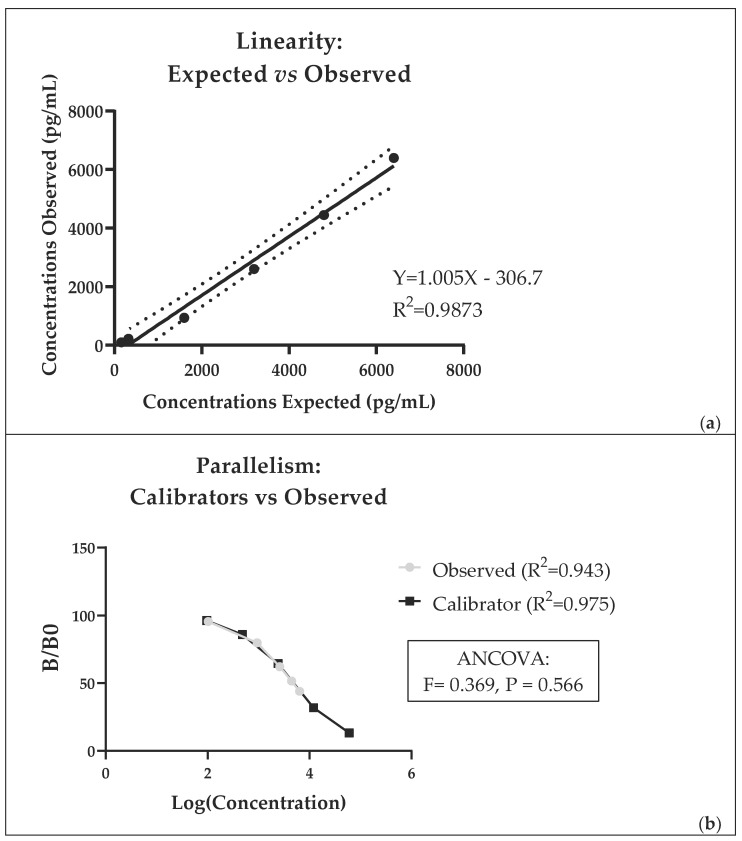
Linearity (panel (**a**); relationship between expected and observed concentrations) and parallelism (panel (**b**); comparison between calibration curve and serial dilutions of a pooled sample) of the ELISA for DHEA-S. In panel (**a**), observed concentrations are shown as black circles. The solid line represents the linear regression, and the dotted lines indicate the 95% confidence bands. Notably, the ELISA kit shows substantial cross-reactivity with DHEA; therefore, the measured concentrations likely reflect the combined contribution of both DHEA-S and DHEA.

The ELISA for DHEA-S showed a strong linear relationship across the analytical range (R^2^ = 0.9873), with a regression slope close to 1 and no significant deviation from linearity (Runs test, *p* = 0.300; Figure 2a), indicating good assay linearity. Regarding ELISA parallelism, both the calibrator curve (R^2^ = 0.975) and the sample dilution curve (R^2^ = 0.943) showed a good fit, with no significant difference between slopes (F = 0.369, *p* = 0.566) (Figure 2b).

### 3.3. Relationship, Agreement, and Ratio Between DHEA and DHEA-S Concentrations

A weak positive association was observed between DHEA concentrations measured by RIA and DHEA-S concentrations measured by ELISA (ρ = 0.220), although this relationship did not reach statistical significance (*p* = 0.097).

**Figure 3:**
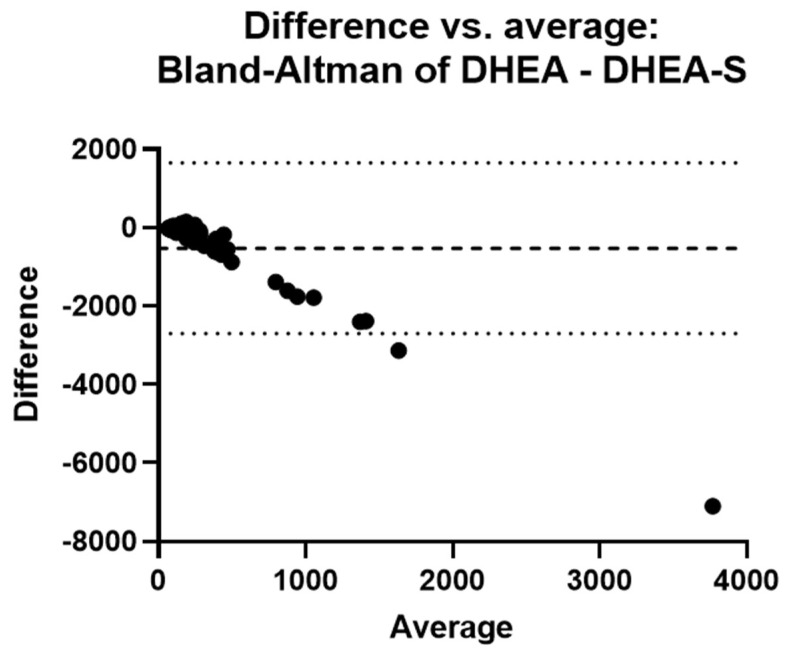
Bland–Altman plot showing agreement between DHEA concentrations measured by RIA and DHEA-S concentrations measured by ELISA. The dashed line represents the mean difference (bias = −525.8 pg/mL), and the dotted lines represent the 95% limits of agreement (−2705 to 1654 pg/mL).

Agreement between DHEA and DHEA-S, evaluated using Bland–Altman analysis, showed a mean difference (bias) of −525.8 pg/mL, indicating that DHEA concentrations were generally lower than DHEA-S values (Figure 3).

Only eight samples (with very low concentrations) had values of DHEA-S lower than DHEA. However, the wide limits of agreement (−2705 to 1654 pg/mL) and the high standard deviation of the bias (SD = 1112 pg/mL) indicate substantial variability between the two measurements.

A trend toward increasing differences with increasing mean concentrations was observed, suggesting the presence of proportional bias. The DHEA/DHEA-S ratio (n = 58) was 0.64 ± 0.11.

## 4. Discussion

To our knowledge, this is the first study reporting circulating DHEA(S) concentrations in broiler chickens.

By both assays, most sample concentrations were close to the lower end of the calibrator’s range. In particular, 15% of samples analyzed with the DHEA-S ELISA were below the detection limit. Similar findings have been reported in wild bird species. For example, Landys et al. [46], using the DHEA RIA kit DSL-8900 in the European nuthatch (*Sitta europaea*), found that 48.5% of samples during the breeding season and 46.2% during the non-breeding season were below the assay detection limit. Likewise, Hau and Beebe [37] reported low detectability of DHEA in the spotted antbird (*Hylophylax naevioides*), with only 25% and 50% of samples exceeding the detection limit during the early and middle breeding seasons, respectively. These observations suggest that circulating concentrations of adrenal androgens may naturally occur at very low levels in some avian species and physiological conditions. Since samples below the detection limit could not be reliably quantified, they were excluded from the statistical analyses. As a consequence, the reported mean DHEA-S concentration may be slightly overestimated. Despite the low concentrations measured in the present study, the analytical validation demonstrated satisfactory performance of both assays. Precision showed acceptable values, the dilution tests demonstrated very good linearity with the expected values, and the dilution curves were parallel to the standard curves. Accuracy of the DHEA RIA, measured by RRs, was, on average, very good. However, excellent RR was obtained at concentrations above 10 ng/mL, whereas at lower concentrations, RRs exceeded 120%. Accuracy and RRs of the DHEA-S ELISA were always excellent.

Concentrations measured by different methods are not directly comparable; nonetheless, the results obtained in the present trial indicate that in this avian species, circulating levels of the two hormone forms are low and of the same order of magnitude (expressed in pg/mL). Concentrations measured by the DHEA-S ELISA kit were generally higher than DHEA, and the Bland–Altman analysis confirmed this pattern by showing a negative bias. This was expected, given the cross-reactivity of the DHEA-S ELISA kit with DHEA, meaning that the concentrations obtained with the ELISA kit may partially represent the combined contribution of both DHEA and DHEA-S. Consequently, the DHEA-S values measured by the ELISA kit may be partially overestimated due to cross-reactivity with DHEA, and the apparent predominance of DHEA-S may, therefore, reflect, at least in part, a methodological effect rather than a true biological difference. Therefore, the analyte measured by the ELISA kit may be more appropriately referred to as “DHEA(S)” rather than DHEA-S alone. However, the wide limits of agreement, the high variability, and the low correlation coefficient indicate poor agreement between the two measures. The increasing difference observed at higher mean concentrations suggests the presence of a proportional bias, which may also reflect concentration-dependent cross-reactivity effects and/or differences in assay performance. In a few samples with very low concentrations, finally, DHEA-S resulted in lower values than DHEA, which is not readily explained. Such findings further encourage the use of utmost caution when looking at the absolute values obtained by different methods. In this case, the concentrations obtained by RIA near the detection limit are likely overestimated, as also suggested by the rather high recovery rates obtained.

DHEA(S) blood concentrations are particularly high in primates (nMol and µMol for DHEA and DHEA-S, respectively), while in other mammals (rat, mouse, rabbit, dog, pig, cattle, sheep, horse) are low or undetectable [4]. The available literature indicates many differences in the origin, synthesis, and sulfation activity, depending on species, sex, and season. In addition, the physiological regulation of circulating DHEA(S) appears complex and species-dependent. Although DHEA secretion is generally considered to be under HPA axis control, responses to ACTH stimulation and acute stressors vary considerably. In mammals, both increases and absent responses to ACTH challenge have been reported [54,55,56,57]. Regarding birds, administration of exogenous ACTH failed to significantly alter DHEA levels in Northern Cardinal [47], and there is general agreement that acute stress (30–60 min restraint) does not affect DHEA concentration in the peripheral circulation (brachial vein) [29,32,35,44,50]. On the other hand, in jugular blood, acute stress induced a significant decrease in DHEA concentrations during the breeding season and an increase during the molting season [32,33,35,47]. These findings suggest that both the regulation and the origin of circulating DHEA(S) remain incompletely understood and may differ substantially among taxa. Considering the age and sex of the birds in the present work, the contribution of the gonads to circulating androgens should be negligible, so the hormones measured were likely from adrenal origin.

Simultaneous measurements of DHEA and DHEA-S blood concentrations have been rarely reported. In mammals, DHEA-S levels were hundreds of times higher than DHEA in humans [14] and tens of times higher than DHEA in narwhals [58]. Conversely, in an elderly study [59], plasma DHEA was higher and DHEA-S lower in various other mammalian species than in humans. Afterward, it was confirmed that, also in cows, circulating DHEA-S is significantly lower than DHEA [55]. The only study investigating both DHEA and DHEA-S in birds, conducted in Japanese quail [28], reported average DHEA-S concentrations similar to or slightly lower than DHEA concentrations. In the present study, concentrations measured by the DHEA-S ELISA kit showed marked inter-individual variability. Similar variability has been reported in cattle, where both concentrations and secretory patterns of these hormones differ substantially among individuals [55], and in humans, where both basal and ACTH-stimulated DHEA levels exhibit considerable inter-subject variability [60]. Furthermore, in the present trial, the relationship between DHEA and DHEA-S concentrations was also highly variable. This variability, together with the lack of correlation and agreement between the two measures, may reflect the dynamic and reversible interconversion between DHEA and DHEA-S [1]. The increasing divergence at higher concentrations further suggests that the relationship between the two measures is not constant across the measurement range, although it is not possible to distinguish whether this reflects underlying physiological regulation or concentration-dependent differences in assay performance.

Regarding birds, we found only one paper in which DHEA-S was assayed [28]. The plasma levels reported in Japanese quail, determined by RIA, were, on average, higher than those of the present work, while showing a narrower range [28].

DHEA values of the present work were of the same order of magnitude as those reported for several wild avian species (Table 1). Nonetheless, comparison of mean concentrations reveals considerable differences among studies and species within the avian class. Future studies are needed to determine whether such differences reflect species-specific characteristics or methodological and biological factors, including assay type, sex, age, season, physiological state, sampling procedures, and sampling site (e.g., brachial vs. jugular vein). Several of these factors have already been identified as potential sources of variation in avian DHEA concentrations (Table 1).

The same ELISA kit for DHEA-S was previously used to measure feather extracts from 40 broiler chickens, yielding concentrations of 125 ± 7 pg/mL or 78 ± 4 pg/mm, about 20 times higher than those of feather corticosterone [26]. It is noteworthy that in plasma, the relationships of the adrenal steroid concentrations are opposite. In the 40 animals identified in the present trial, plasma corticosterone concentrations were assayed in our previous study [27], yielding averages 151 and 74 times higher than those found in the present trial for DHEA and DHEA(S), respectively. Overall, these findings highlight a marked divergence in steroid profiles between feather and plasma matrices.

Some limitations of the present study should be acknowledged. Although both assays showed satisfactory analytical performance, their sensitivity was not optimal for all samples, with a proportion of DHEA-S concentrations falling below the detection limit. Since values below the detection limit could not be reliably quantified, they were excluded from the statistical analyses. This approach may have resulted in a slight overestimation of the mean DHEA-S concentration. However, assigning arbitrary values to these samples would have introduced an additional source of uncertainty and could have affected the reliability of the analytical validation results, particularly those related to linearity and agreement between methods. In addition, the substantial cross-reactivity of the DHEA-S ELISA antibody with DHEA complicates the interpretation of the measured values and may partially contribute to the apparent predominance of DHEA-S over DHEA. Consequently, caution is required when interpreting absolute concentrations and the relationship between the two hormones.

## 5. Conclusions

The two methods for the determination of DHEA and DHEA-S were evaluated in plasma samples using direct assays (i.e., without prior extraction), with the aim of developing faster and simpler analytical procedures. Repeatability (precision) was acceptable. Accuracy (recovery rates) and dilution tests (parallelism and linearity) were very good, indicating that both methods provided acceptable analytical performance and may be useful for comparative studies among samples.

In general, the sensitivity of both methods appears sufficient but suboptimal for chicken plasma, as some samples fall below the detection limit with the ELISA kit. This may be overcome by setting up an efficient extraction procedure that enables sample concentration. The present study provides preliminary information on circulating DHEA and DHEA-S concentrations in this avian species. Both hormones were detected at generally low concentrations and within a similar order of magnitude. Despite the analytical constraints discussed above, the methods proved suitable for the quantification of DHEA(S) in chicken plasma and may represent useful tools for future studies investigating endocrine indicators of stress and welfare.

Future studies combining immunoassays with more specific analytical techniques may provide a more detailed characterization of circulating DHEA and DHEA-S concentrations in avian species. Moreover, it will be interesting to study the relationships among concentrations across different matrices (plasma, feathers, muscle), which could indicate distinct time-dependent patterns of hormone deposition at the tissue level. As applied to other species, the concentrations of DHEA(S) can be appropriately integrated with those of glucocorticoids. The advisability of these measures can provide new insights into HPA activity and may act as a further physiological indicator available for research in the field of animal stress and welfare assessment.

## Figures and Tables

**Table 1:** Avian species in which plasma DHEA(S) concentration has been assayed. All the values are referred to DHEA, except ^⮾^, which are DHEA-S concentrations. All the assays are RIA, except ^‡^, which are ELISA. References with * utilized the RIA kit DSL-8900.

Species	References	Values (Approximate Ranges)
**Japanese quail (*Coturnix japonica*)**	Tsutsui and Yamazaki, 1995 [28]	^⮾^ 1.5–2.5 ng/mL 1–4 ng/mL
**Song sparrow** **(*Melospiza melodia*)**	Soma and Wingfield, 2001 [29]	0.3–0.9 ng/mL
	Soma et al., 2002 [30]	0.1–3 ng/mL
	* Goodson et al., 2005 [31]	600–1500 pg/mL
	* Newman et al., 2008 [32]	300–1200 pg/mL
	* Newman and Soma, 2009 [33]	200–1100 pg/mL
	* Newman et al., 2010 [25]	1–5 ng/mL
	* Maddison et al., 2012 [34]	1–1.5 ng/mL
	* Newman et al., 2013 [35]	0.7–2.8 ng/mL
**Spotted antbird** ***(Hylophylax naevioides)***	Hau et al., 2004 [36]	0.2–1.5 ng/mL
	Hau and Beebe, 2011 [37]	0.2–1.5 ng/mL
**Domestic geese (*Anser domesticus*)**	Xuan et al., 2005 [38]	70–1000 pg/mL
**White-throated sparrow** **(*Zonotrichia albicollis*)**	Spinney et al., 2006 [39]	0.1–2.5 ng/mL
**Buff-breasted wren** **(*Thryothorus leucotis*)**	Gill et al., 2008 [40]	0.25–1.7 ng/mL
**Song wren** **(*Cyphorhinus phaeocephalus*)**	Bush et al., 2008 [41]	0.3–0.6 ng/mL
**European starling (*Sturnus vulgaris*)**	* Chin et al., 2008 [42]	600–2400 pg/mL
	Pintér et al., 2011 [43]	0.4–0.9 ng/mL
**Brent geese (*Branta bernicla bernicla*)**	Poisbleau et al., 2009 [44]	0.1–0.2 ng/mL
**Zebra finch (*Taeniopygia guttata*)**	* Taves et al., 2010 [45]	1.5–4 ng/mL
**European nuthatch (*Sitta europaea*)**	* Landys et al., 2013 [46]	200–400 pg/mL
**Northern cardinal** **(*Cardinalis cardinalis*):**	^‡^ Fokidis, 2016 [47]	0.5–2.5 ng/mL
**Manakin (*Manacus vitellinus*)**	* Eaton et al., 2018 [48]	1–2 ng/mL
**Painted bunting (*Passerina ciris*)**	Rohwer et al., 2020 [49]	0.1–0.6 ng/mL
**Barnacle geese (*Branta leucopsis*)**	^‡^ Doyle et al., 2021 [50]	0.10–4.65 nmol/L

**Table 2:** Recovery rates (RRs) of the added DHEA hormone to a pooled sample (RIA kit).

Volumes (µL)		Concentrations (pg/mL)		
Pooled Sample (390 pg/mL)	Standard (42,000 pg/mL)	Observed	Expected	RR (%)
196	4	1490	1222	122
190	10	3040	2470	123
180	20	5660	4551	124
150	50	10,900	10,792	101

**Table 3:** Recovery rates (RRs) of the added DHEA-S hormone to a pooled sample (ELISA kit).

Volumes (µL)		Concentrations (pg/mL)		
Pooled Sample (303 pg/mL)	Standard (60,000 pg/mL)	Observed	Expected	RR (%)
195	5	1666	1794	93
190	10	3495	3286	106
180	20	6397	6271	102
160	40	11,600	12,241	95

## Data Availability

Data are available from the corresponding authors upon request.
